# Barriers and Enablers of Older Patients to Deprescribing of Cardiometabolic Medication: A Focus Group Study

**DOI:** 10.3389/fphar.2020.01268

**Published:** 2020-08-20

**Authors:** Stijn Crutzen, Gert Baas, Jamila Abou, Tessa van den Born-Bondt, Jacqueline G. Hugtenburg, Marcel L. Bouvy, Mette Heringa, Katja Taxis, Petra Denig

**Affiliations:** ^1^Department of Clinical Pharmacy and Pharmacology, University Medical Center Groningen, University of Groningen, Groningen, Netherlands; ^2^SIR Institute for Pharmacy Practice and Policy, Leiden, Netherlands; ^3^Department of Clinical Pharmacology and Pharmacy, Amsterdam UMC, location VUMC, Amsterdam, Netherlands; ^4^Division of Pharmacoepidemiology and Clinical Pharmacology, Utrecht Institute for Pharmaceutical Sciences, Utrecht University, Utrecht, Netherlands; ^5^Unit of PharmacoTherapy, Epidemiology and Economics, Groningen Research Institute of Pharmacy, University of Groningen, Groningen, Netherlands

**Keywords:** primary care, deprescribing, cardiometabolic medication, patient’s perspective, aged

## Abstract

**Background:**

Deprescribing has been recommended for managing polypharmacy but deprescribing preventive medication in older patients is still uncommon. We aimed to investigate older patients’ barriers to and enablers of deprescribing cardiometabolic medication.

**Methods:**

Two focus groups were conducted among patients ≥70 years with polypharmacy, including cardiometabolic medication. Purposive sampling through four community pharmacies was used in two regions in the Netherlands. A topic list was developed using literature and the theoretical domains framework (TDF). The meetings were audio recorded, transcribed verbatim and coded using thematic coding, attribute coding and the TDF. In addition, patients were categorized on attitudes towards medication and willingness to stop.

**Results:**

The meetings were attended by 17 patients and 1 caregiver (71 to 84 years). In total 15 barriers and 13 enablers were identified within four themes, partly related to beliefs, fears and experiences regarding using or stopping medication, and partly related to the relationship with the health care professional and the conditions to stop. Some patients attributed their wellbeing to their medication and were therefore unwilling to stop. Reducing cardiometabolic medication because of less strict treatment targets confused some patients and was a barrier to deprescribing. Having options to monitor clinical measurements and restart medication were enablers. Patients were only willing to stop cardiometabolic medication when this was proposed by a HCP they trusted. Patients with a positive attitude towards medication varied in their willingness to stop cardiometabolic medication. Patients with a negative attitude towards medication were generally willing to stop medication but still perceived several barriers and may consider some medication as being essential.

**Conclusion:**

Fears, beliefs, and experiences regarding using and stopping medication as well as trust in the HCP influence willingness to have medication deprescribed. Attitudes towards medication in general do not necessarily translate into willingness or unwillingness to stop specific medication. For deprescribing cardiometabolic medication, patient involvement when setting new treatment targets and monitoring the effects on short-term outcomes are important.

## Introduction

Intensive glucose and blood pressure lowering treatment can prevent long-term complications in people with diabetes and/or hypertension but it may come with an increased risk of harm (The Diabetes Control and Complications Trial Research Group, 1993; Gerstein et al., 2008; Wright et al., 2015). Whether in older patients intensive glycemic and blood pressure control is more beneficial than harmful has been a matter of debate (Bejan-Angoulvant et al., 2010; Cushman et al., 2010; Gerstein et al., 2011; Sue Kirkman et al., 2012; Vijan et al., 2014; Beddhu et al., 2018; Williamson et al., 2019). While some investigators argued that intensive treatment should be maintained in older patients because of shown benefits in clinical trials, others reasoned that the limitations of these trials resulted in overestimating benefits and underestimating harms, particularly for older patients (Scott et al., 2019; Supiano and Williamson, 2019). In addition, it has been stated that in people with a reduced life expectancy the benefits of preventive cardiometabolic medication become less relevant (Holmes et al., 2006; Maddison et al., 2011). Given these considerations, it has been recommended to consider deprescribing such medication in older or frail people with diabetes and/or hypertension (Primary Health Tasmania; Farrell et al., 2017). Deprescribing is the process of stopping or reducing of medication by a health care professional (HCP) in consultation with the patient, with the goal of managing polypharmacy and improving patient outcomes (Woodward, 2003; Reeve et al., 2015). Despite initiatives in various countries to support healthcare providers to deprescribe medication in older patients (The Bruyère Deprescribing Research Team; Vimalananda et al., 2017; Seidu et al., 2019), it seems that deprescribing of glucose-lowering and antihypertensive medication is uncommon (Sussman et al., 2015; McAlister et al., 2017). It might be that patients have not been sufficiently involved in this process.

Previous qualitative research identified multiple barriers to deprescribing in primary care (Schuling et al., 2012; Anderson et al., 2014; Ailabouni et al., 2016; Wallis et al., 2017; Gillespie et al., 2018). One of the barriers expressed by general practitioners (GPs) is the unwillingness of patients or their relatives to discontinue medication (Schuling et al., 2012; Anderson et al., 2014; Ailabouni et al., 2016; Wallis et al., 2017; Gillespie et al., 2018). GPs believe that patients may be resistant towards deprescribing and they fear that patients feel given up on when medication is stopped (Gillespie et al., 2018; van Middelaar et al., 2018). This perceived unwillingness is in contrast with results from survey studies in which a large proportion of patients stated that they were willing to stop one or more medications when advised to do so by their physician (Reeve et al., 2013b; Sirois et al., 2017; Reeve et al., 2018; Schiøtz et al., 2018). Enablers of deprescribing for patients are a general dislike of medication, the feeling that the medication is not needed anymore and fear of side effects and interactions (Reeve et al., 2013a; Luymes et al., 2016; Reeve et al., 2016; Gillespie et al., 2018). Patients are more willing to stop when they trust their physician, when it is conducted as a test and monitoring is offered (Reeve et al., 2013a; Luymes et al., 2016; Reeve et al., 2016; Gillespie et al., 2018). An important barrier for patients is the belief that the medication is still needed or that they may have benefits in the future (Reeve et al., 2013a; Luymes et al., 2016; Reeve et al., 2016; Gillespie et al., 2018).

Research on patients’ barriers to and enablers of deprescribing in both older and younger populations has mainly been focused on medication in general or on symptomatic medication that can be considered inappropriate to take for an extended period of time, such as benzodiazepines, antidepressants and proton pomp inhibitors (Reeve et al., 2013a). Patients’ barriers to deprescribing and their willingness to stop medication may be different for preventive cardiometabolic medication. One study among relatively young patients showed that there were several medication specific beliefs and experiences towards deprescribing of cardiovascular medication (Luymes et al., 2016). Whether this is also the case in older people has not been explored so far. The aim of the present study was to investigate older patients’ barriers to and enablers of deprescribing cardiometabolic medication.

## Methods

### Design and Setting

Two focus groups were performed to identify barriers to and enablers of deprescribing cardiometabolic medication among older patients with polypharmacy. The focus groups were held in November 2018 in a community pharmacy and in a primary health care center in different regions of The Netherlands. The focus groups were moderated by JH (PharmD, PhD) and MH (PharmD, PhD), both are female senior researchers with a broad interest in optimizing pharmacotherapy and pharmacy-based interventions. Both researchers have experience with moderating focus groups and received interview training in the past. There was no prior relationship with the focus group participants. The focus groups were attended by the participants and the moderator and two junior researchers (GB and SC). Demographic information was collected using a short questionnaire. Caregivers were asked to fill in the information about their care receiver. Patients were asked to bring a list of their medication to facilitate discussion about specific medication. All procedures performed in this study involving human participants were in accordance with the ethical standards of the Medical Ethic Commission of VU Medical Center (FWA00017598) and with the 1964 Helsinki declaration and its later amendments.

### Participants and Recruitment

Purposive sampling was used, where participants were recruited through four community pharmacies, situated in an urban area, of which two were in a large city and two were in a large town. Eligible patients were identified from the community pharmacy information systems. This was done to ensure that the participants used the medication of interest, were managed by different HCPs and came from different settings. In total 120 eligible patients received an invitation letter. Known caregivers for eligible patients were invited directly and patients were informed in the invitation letter that they could send their caregiver to the focus group. Patients could contact their community pharmacy or one of the researcher by phone or email when they wanted to participate. Patients who did not respond to the letter within 1 week were called to ask whether they were willing to participate until sufficient patients agreed to participate. The aim was to invite 10 participants per focus group with the expectation that between 7 and 10 would actually be able to participate. Informed consent was collected from the patients and caregivers who attended the focus groups. Participants were compensated for their time with a twenty euro gift card.

#### Inclusion Criteria (Based on the Community Pharmacy Information System)

- Older patients: At least 70 years of age
- Polypharmacy: Use of 5 or more different medications at Anatomical Therapeutic Chemical (ATC) level 5 (World Health Organization, 2019), where chronic was defined as three or more dispensings in the past year, of which at least 1 in the last 6 months
- All of the patients were part of one subgroup and at least two patients from each subgroup per focus group were included:
  - Subgroup cardiovascular without diabetes: at least two unique cardiovascular medications (ATC code C) dispensed between 1 July and 31 October 2017 and between 1 July and 31 October 2018, without any dispensing for diabetes medication (ATC A10) in 1 July and 31 October 2018
  - Subgroup type 2 diabetes: at least one noninsulin diabetes medication (ATC code A10B) dispensed in July–October 2017 and between 1 July and 31 October 2018

#### Exclusion Criteria (According to the Assessment of the Patients’ Community Pharmacist)

- Too frail/ill: Patient was too frail/ill to be approached and no caregiver was known
- Unwilling to participate in research: Patient and/or caregiver did not wish to be approached for research
- Non-Dutch speaking: Patient and/or caregiver did not speak Dutch

### Topic List

A topic list was developed using existing literature on barriers to and enablers of deprescribing (Reeve et al., 2013a; Galazzi et al., 2016; Luymes et al., 2016; Palagyi et al., 2016; Reeve et al., 2016; Sirois et al., 2017; Reeve et al., 2018; Weir et al., 2018) and the theoretical domains framework (TDF) (Michie, 2005; Atkins et al., 2017). The TDF describes important factors underlying behavioral change and implementation issues. This framework can be helpful in identifying behavioral domains that are relevant for patients in relation to the implementation of deprescribing and their willingness to stop medication. It includes the following domains: knowledge, skills, social/professional identity, beliefs about capabilities, beliefs about consequences, motivation and goals, memory attention and decision processes, environmental context and resources, social influences, emotion, behavioral regulation, and nature of the behaviors. The topic list was piloted separately by the two moderators. As a result, the introduction of the topic list and the wording of some questions were changed. A translated version of the topic list can be found in “**Appendix I**”. After a short introduction on the topic, the participants were asked to introduce themselves and to elaborate on the medication they were taking. Next, the participants were asked about their experiences with stopping medication, their opinion about stopping medication and their considerations for wanting to continue or stop specific medication. Also, they were asked under which conditions and under whose supervision they would be willing to stop medication. Finally, they were given the opportunity to raise other issues they felt were relevant that had not yet been addressed.

### Analyses

The focus groups were audio recorded and transcribed verbatim. Field notes were used to enhance the transcripts with nonverbal information. ATLAS.ti version 5.2.18 was used for the analysis. Directed content analysis was conducted in order to identify barriers and enablers to deprescribing. Two researchers (SC and TB) developed a coding scheme prior to coding the focus groups (**Appendix II**). The following levels of coding were used: (1) thematic codes coding barriers and enablers to deprescribing, (2) the twelve domains of the TDF, (3) attribute codes for descriptive information about what was stated. Attribute codes were added as needed in an iterative process. This coding scheme was discussed with PD and KT. Quotes were coded by SC and TB separately, and consensus was reached by discussing disagreements in coding. The coded transcripts were analyzed in two ways.

1. Quotes coded as barriers to or enablers of deprescribing were sorted, summarized and reduced by two researchers (SC, GB). The quotes were sorted per domain from the TDF, subdivided in barriers and enablers. Afterwards, overarching themes were identified within the barriers and enablers. Consensus was reached by discussing disagreement, remaining disagreement was discussed with a third researcher (PD). Example quotes were selected for the manuscript.
2. Barrier and enablers were extracted per patient and a summary of these barriers and enablers was made per patient (**Appendix III**). Patients were then categorized according to their attitudes towards medication (positive, negative, indifferent) and their willingness to stop medication (resistant, willing, indifferent), based partly on the typology of Weir et al. (2018). This categorisation was conducted by SC and PD independently, and consensus was reached by discussing disagreements.

Quotes were translated from Dutch to English for this article by SC and the translations were checked by PD and KT. No feedback from the participants was collected. The Consolidated Criteria for Reporting Qualitative Research (COREQ) was used to ensure completeness of reporting (Tong et al., 2007).

## Results

### Characteristics of Participants

In total 17 patients and 1 caregiver out of the 120 invited by mail participated in the focus groups. Reasons for not wanting to participate were: not interested to participate, illness, patient felt that he/she could not contribute anything relevant, and/or other obligations. The age of the patients ranged from 71 to 84 years. Most patients took medication for cardiovascular disease and half of them for type 2 diabetes (**Table 1**). In total 15 barriers and 13 enablers were identified within four major themes (**Table 2**), including “Opinions and beliefs about medication” and “Opinions and beliefs about stopping medication,” “Relationship with the health care professional,” and “Conditions to stop.” Barriers and/or enablers were identified for 7 of the 12 domains of the TDF (**Table 2**).

**Table 1:** Characteristics of the patients.

	Focus group 1	Focus group 2
Setting	Large city	Large town
Number of participants	8	10
Male patients	5	6
Age of patients (median, range), years	78 (74–82)	77.5 (71–84)
Patients with type 2 diabetes	5	4
Patients with cardiovascular disease	7	10
Patients with 5-10 medications^a^	6	4
Patients with >10 medications^a^	2	5

The demographic information of the care receiver was used for this table.

^a^For one participant the number of medications was missing.

**Table 2:** Barriers and enablers subdivided in four themes.

Barriers theme 1: Opinions and beliefs about medication	TDF domain
**B1:** Medication is seen as essential because of knowledge or beliefs about the underlying disease	Knowledge/Beliefs about consequences
**B2:** Belief that he/she is doing well, because of the medication	Beliefs about consequences
**B3:** Belief that statin is needed because cholesterol is successfully lowered	Knowledge
**B4:** Confusion about treatment targets for glucose and cholesterol	Knowledge
**B5:** It is hard to distinguish between the effects of different medications	Knowledge
**Enablers theme 1: Opinions and beliefs about medication**	**TDF domain**
**E1:** Belief that medication is bad for long-term health	Beliefs about consequences
**E2:** Fear/belief that medication can cause severe side effects	Emotion/Beliefs about consequences
**E3:** It is unclear why statins are needed when cholesterol has always been low	Knowledge
**E4:**Taking a lot of medication is a burden	Motivation and goals
**E5:** Would like to take fewer medications in general	Motivation and goals
**E6:** Experienced side effects	Nature of behavior
**Barriers theme 2: Opinions and beliefs about stopping medication**	**TDF domain**
**B6:** Belief that it can be dangerous to stop medication	Beliefs about consequences
**B7:** Fear about what will happen when you stop medication	Emotion
**B8:** Bad experiences with stopping medication	Nature of behavior
**B9:** Unwilling to change medication when satisfied with their current health status	Nature of behavior
**Enablers: Theme 2: Opinions and beliefs about stopping medication**	**TDF domain**
**E7:** Belief that it is good to change medication to prevent becoming dependent	Beliefs about consequences
**E8:** Feeling good, therefore willing to stop medication	Motivation and goals
**E9:** Good experiences with stopping medication	Nature of behavior
**Barriers theme 3: Relationship with the health care professional**	**TDF domain**
**B10:** Dependence on the physician to stop medication, since the patient lacks the knowledge to make such decisions	Knowledge/Beliefs about capabilities
**B11:** Distrust other HCP than his/her prescribing physician to change medication	Emotion
**B12:** The prescribing physician does not have the time to discuss stopping medication	Environmental context and resources
**B13:** The prescribing physician does not take medication related complaints seriously	–
**B14:** Distrusts the nurse practitioner to change medication	Emotion
**B15:** Distrusts the community pharmacist to change medication	Emotion
**Enablers theme 3: Relationship with the health care professional**	**TDF domain**
**E10:** Willingness to stop medication when their physician proposes to do so, since the physician knows more about medication	Knowledge/Beliefs about capabilities
**E11:** Positive attitude towards community pharmacist giving advice on which medication might be stopped during a medication review	Knowledge
**Enablers theme 4: Condition to stop**	**TDF domain**
**E12:** Clinical measurements are used to monitor after medication has been stopped	–
**E13:** Stopping medication is considered a test/Medication can be restarted	–

TDF, theoretical domains framework.

### Themes 1 and 2: Opinions and Beliefs About Medication and Stopping Medication

Patients’ opinions about their medication strongly influenced their willingness to stop medication. There were some patients who attributed their wellbeing to their medication and were therefore in general unwilling to stop (**B2**). In many cases patients talked about medication in general terms. They were content with their overall health (**B9**), so they did not see a reason to change any medication.

**FG1, P6 (B9):** *“I have had them for years, and I have no complaints. The other day they mentioned it [stopping medication] again, this diabetes sugar person [specialised nurse practitioner who provides diabetes care for this patient]. She said: “shall we adjust the medication”, I said: I wouldn’t do it”*

Some patients were more explicit about the necessity of specific medication, referring to the severity of the underlying disease and the consequences of stopping that medication (**B1**).

**FG2, P5 (B1):** *“I have been taking blood thinners for years. When you have had a TIA (transient ischemic attack) and you would stop with the blood thinners, then you run the risk of it happening again. So you will not stop, no”*.

One participant came to the opposite conclusion. This participant expressed that he/she was doing well and was therefore willing to stop medication (**E8**).

Several patients had a negative opinion about their medication, often because of side effects. Experiencing side effects was a reason for wanting to stop medication (**E6**), in particular related to statins. Also, fear of developing side effects was a reason for wanting to stop (**E2**). This was expressed both in general and more specific terms. Some participants mentioned fear of specific side effects of particular medication, whereas others sometimes regarded medication as “poison” or not good for your health (**E1**) or were afraid to become dependent on their medications (**E7**).

**FG1, P2 (E1):** *“Well, I think that if you are taking medication for too long, that this is not good for your body”*.

Some patients wanted to take less medication in general (**E5**). Having to take a large amount of medication was seen as burden (**E4**). Confusion about clinical measurements like blood pressure, glucose and cholesterol, and conflicting treatment targets mentioned by HCPs could lead to resistance to stop medication (**B4**).

**FG1, P4 (B4):** *“Well, my husband has been using metformin for years. At a certain point he lost some weight, and what do you know, she [specialised nurse practitioner] said it’s not needed anymore. So I started to think, how high is the sugar allowed to go? Right, cause somehow I didn’t really trust it. And then he [GP] said: “well, it’s fine as long as the sugar doesn’t go above 8. Below 8, and it’s all fine”. How is that possible, 6 years, 6 years ago they said it is not allowed to be above 5. (…) Then you do not understand, that suddenly it can go away. That you do not have to use it anymore”*.

Some patients did not seem to understand why a statin was needed, particularly when the statin was prescribed for primary prevention. Low cholesterol levels led to opposing views among patients. Several patients wondered why they took statins when their cholesterol was already low (**E3**), whereas another patient did not see a reason to stop since the statin was successfully lowering the cholesterol (**B3**). Uncertainty and fear about what would happen when medication is stopped or previous bad experiences with stopping were mentioned as reasons for not wanting to stop (**B6, B7, B8**). In contrast, other patients referred to good experiences with stopping (**E9**).

### Themes 3 and 4: Relationship With Health Care Professional and Conditions to Stop

In general, patients were only willing to stop cardiometabolic medication when this was proposed by a HCP they trusted. When this was proposed by another than their prescribing HCP they would be less willing to stop, for example, when a GP would propose to stop treatment that was prescribed by their specialist (**B11**). On the other hand, even patients with a positive opinion about their medication may be willing to stop medication when their physician would propose to do so (**E10**). Important for having trust in the HCP was the perception that the HCP was knowledgeable about the medication and the underlying disease. Most often the trusted HCP was a prescribing specialist or GP. In general, patients were negative about initiation of deprescribing by a nurse practitioner or a community pharmacist (**B14, B15**). Then again, patients who stated that they had received a medication review in the past led by a community pharmacist were more positive about an advisory role of the pharmacist (**E11**).

**FG2, P10 (E10):** [What if a physician would propose to stop medication?] *“Well, he [physician] knows more about it [medication] than me, so I would trust him, if later on you don’t trust it you can always come back to it”*

Some participants stated that they lacked knowledge to decide about stopping medication. This lack of knowledge was relevant for patients with a negative opinion about their medication and wanting to stop medication, as well as for patients with a more positive opinion about their medication. For patients with a negative opinion about their medication, a lack of knowledge was seen as a barrier to stop medication (**B10**). So even if they were willing to stop, they felt it was only possible when their physician endorsed it.

**FG1, P2 (B10):** *“Of course you can argue with your doctor but because he has learned for it, I think you will always end up with the short end of the stick”*

Patients also felt that their physician sometimes lacked time (**B12**), and did not always take their complaints serious (**B13**), both of which hindered having a conversation about stopping medication. Participants were more comfortable to stop medication when stopping was considered a test (**E13)** and when this process was monitored using, for example, clinical measurements (**E12**).

**FG2, P1 (E12):** *“That they take your experiences after stopping into account and if possible do some blood tests or I don’t know, whatever they can measure.’’*

### Patient Typology

**Figure 1:**
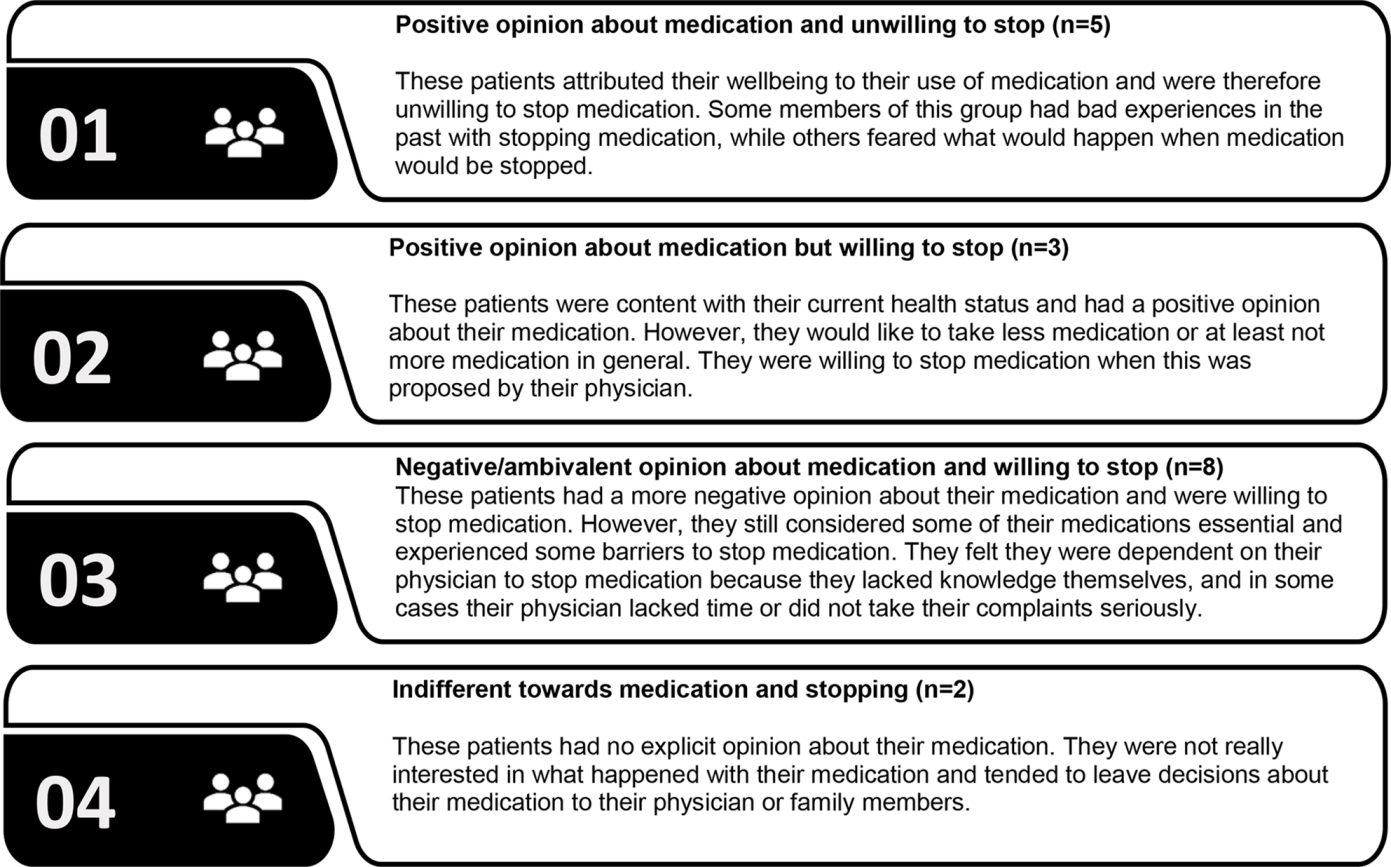
Patients’ typology based on attitudes towards medication and willingness to stop medication.

Four typologies were identified based on the participants’ attitudes towards medication and their willingness to stop medication. The first 2 types had a positive opinion about their medication, but differed in willingness to stop medication (**Figure 1**). People belonging to the third type had a more negative opinion about their medication and were willing to stop medication but still considered some medication essential. The fourth type was largely indifferent towards their medication and towards stopping medication (**Appendix III, Table III.1**).

## Discussion

### Summary

Views about medication and about deprescribing strongly varied between and within patients. Fears, beliefs, and experiences regarding using or stopping medication influenced the willingness to have medication deprescribed. A good health status was for some patients a reason to consider stopping and for others a reason not wanting to stop medication. Some patients perceived cardiovascular or diabetes medication as essential to reach their treatment targets or prevent serious events. In other cases, patients perceived less need for medication, particularly for statins. Potential and existing side effects contributed to a negative view on medication and enhanced the willingness to stop certain medication. Patients were more willing to stop medication when this was proposed by a HCP they trusted, which was often the prescribing physician. Although many patients felt they lacked necessary knowledge about their medication, were unfamiliar with deprescribing and considered it as something they would undergo, most of them would like to be involved in the process of deprescribing. Monitoring and the option to restart were considered important conditions for deprescribing. Four general typologies were identified based on the participants’ views about medication and willingness to stop, that is, (1) positive attitude towards their medication and resistant towards stopping; (2) positive attitude towards medication but willing to stop; (3) negative attitude towards medication and willing to stop, and (4) indifferent towards both their medication and stopping of medication.

### Comparison With Existing Literature

#### Treatment Targets and Short-Term Outcomes

By focusing on preventive cardiometabolic medication, we identified both barriers and enablers to deprescribing that were specifically related to treatment targets and short-term outcomes. It seems that patients focus more on short-term than on long-term outcomes of preventive treatment. This has also been observed in a recent interview study on treatment adherence conducted in the United States of America (USA), where almost half of the patients expected only short-term benefits (e.g. low blood pressure or glucose levels) of their hypertension and diabetes medication (Gibson et al., 2018). Previous qualitative research among younger Dutch patients identified well-controlled blood pressure as an enabler to deprescribing of antihypertensive medication (Luymes et al., 2016). An interview study on medication-related concerns among type 2 diabetes (T2D) patients in the USA showed that the continued need for medication when treatment targets were reached may confuse patients (Tjia et al., 2007). Our study showed that having low cholesterol was a reason for wanting to stop medication for some patients but for others low cholesterol was seen as evidence of the efficacy of the treatment, making them reluctant to stop.

#### Patient Involvement

Involving patients in achieving targets when initiating or intensifying treatment may be a barrier for deprescribing cardiometabolic medication at a later stage. Medication can be seen as essential for achieving targets. This notion is supported by an interview study conducted in the USA showing that medication that improved test results is seen as more important by patients, especially when these test results are discussed with the patient (Lau et al., 2008). In the Netherlands, T2D patients receive quarterly and yearly check-ups, in which HbA1c, blood pressure and cholesterol are tested and discussed with the patient (Boels et al., 2017; NHG, 2018). Patients with a long history of diabetes have received years of instruction during these check-ups on the importance of achieving treatment targets by adhering to medication. Re-evaluating these treatment targets, by personalizing targets based on age and diabetes duration has shown to improve cardiometabolic control (Boels et al., 2017). Our study showed that such relaxation of treatment targets can cause confusion for some patients and may lead to reluctance towards stopping medication.

#### Follow Up

The importance of follow up for patients has been emphasized in previous qualitative research (Reeve et al., 2013a; Luymes et al., 2016; Reeve et al., 2016; Gillespie et al., 2018). The use of clinical measurements, such as blood pressure or glucose levels, during follow up is a necessary condition for many patients to evaluate the outcome of deprescribing cardiometabolic medication (Luymes et al., 2016). In addition, closely monitoring clinical outcomes can help in safely tapering off cardiometabolic medication ensuring deprescribing will not jeopardize disease management. This may require additional explanation to patients, since slight increases in these outcomes can be expected and are wanted.

#### Deprescribing Typology

When looking at the willingness to stop medication and attitudes towards medication at individual patient level, different “deprescribing” typologies emerge. Previous Australian qualitative research in older people identified three types of patients when it comes to willingness to have any medication deprescribed, which were characterized as “attached to medication,” “would consider deprescribing” and “defers to others” (Weir et al., 2018). In our study a more nuanced picture was observed. Regarding the first group with a positive attitude towards their medication, who were classified by Weir et al. as being resistant towards deprescribing (Weir et al., 2018), we found that only part of these participants were resistant to deprescribing. Several of them were willing to stop when this was proposed by their physician, emphasizing the influence of the patients’ relationship with their HCP on willingness to undergo deprescribing. For the second group, with a more negative attitude towards their medication it was confirmed that they were indeed willing to stop some medication in general but not always willing to stop with certain medication. Furthermore, while these patients were linked by Weir et al. to being more knowledgeable and preferring an active role in decision making (Weir et al., 2018), this preference for shared decision making was not seen for all patients in our study. Some of them explicitly mentioned having a lack of knowledge and wanting their physician to guide the decision. Although they want their opinions and preferences to be taken into account, they do not necessarily want to make the decision about stopping medication or they belief they are unable to do so. Our study illustrates that a general negative attitude towards medication does not always translate to all medication and that positive attitudes towards medication do not always translate to unwillingness to stop medication. Many factors can contribute to the perceived importance of a certain medication, including perceived benefits and harms, but also a long history of use and trust in the prescribing physician (Lau et al., 2008). Differences in perceived importance between medications within a patient may partly explain the contrasting finding in survey studies that on the one hand patients are satisfied with their current treatment and on the other hand state that they are willing to stop one or more medications when this is proposed by their physician (Reeve et al., 2013b; Sirois et al., 2017; Reeve et al., 2018; Schiøtz et al., 2018). These possible differences in willingness to stop different medication groups both within and between patients should be explored further.

#### Theoretical Domains Framework

The use of the TDF allowed us to describe barriers or enablers to deprescribing from the patients’ perspective in more detail compared to previous studies. Guided by the TDF, we distinguished the subtle but relevant difference between a “belief that something bad will happen” and “fear that something bad may happen,” where previous papers reported only fear of what would happen when medication is deprescribed as a barrier (Reeve et al., 2013a). For some domains of the TDF, no clear barriers or enablers were identified. This may be due to the fact that TDF was developed to assess or describe factors that influence behavioral change (Atkins et al., 2017), whereas patients did not think of themselves as the main actor in the process of deprescribing. Most patients considered their HCP to be responsible and thus considered deprescribing as something they would undergo. Therefore, it is not surprising that no barriers or enablers were mentioned by the participants in the domains “skills,” “memory attention and decision processes.” and “behavioral regulation.” When deprescribing becomes more common in practice and patients may become more active in this decision-making process additional barriers or enablers might be identified in these domains.

### Strengths and Limitations

Two focus groups were conducted including a sample of 17 older adults and 1 caregiver, which were exposed to different HCPs and came from different settings. Caregivers can have a different perspective on medication use and deprescribing than patients themselves. By including a caregiver we strived to include this perspective in our study, but including only one caregiver might not be enough for this purpose. Recruitment of patients was done in two urban areas in the Netherlands, whereas attitudes towards deprescribing might be different in more rural areas or other countries. The exclusion of non-Dutch speaking patients limits the representativeness for minority groups in the Netherlands. Also, selection bias may have been introduced by inviting patient for a focus group about stopping medication, since this may attract relatively well informed patients with a strong opinion on their medication. The results, however, indicate that also patients without strong opinions were included. Most participants had limited experience with deprescribing and eligibility for deprescribing was not assessed. As a consequence some of the questions and topics discussed were hypothetical in nature.

### Implications for Research and Practice

This study expands on the knowledge on attitudes towards deprescribing and willingness to stop medication in older patients. At a patient level, we identified four typologies, namely positive towards medication and unwilling to stop, positive towards medication but willing to stop suggested under certain conditions, negative towards medication and willing to stop certain but not all medication, and indifferent towards medication and stopping. Depending on this typology, HCPs might adapt their strategy to inform patients about the relevance of deprescribing and involve them in the process. Further research is needed to confirm this typology in other settings, and to develop and evaluate tailored strategies for different patient groups. Furthermore, this study provides new insights in the attitudes and willingness to stop cardiometabolic medication. The willingness to stop medication depends on which medication is discussed. Whether or not a patient would be willing to stop certain medication can be based on incorrect interpretation of information by the patient, for example, with regard to treatment targets and the relevance of short-term outcomes. These findings are relevant when developing strategies to support the implementation of deprescribing cardiometabolic medication. Further research is needed on how willingness to stop medication is affected by patients’ expectation about the effects and continuous need of cardiometabolic medication on short- and long-term health and by the way HCPs communicate about these expectations.

For HCPs who want to deprescribe medication, it is important to take the patient’s beliefs into account. Each patient comes with a set of different beliefs about their health and medication. These beliefs result in a set of unique barriers to and enablers of deprescribing (Weir et al., 2018) and need to be identified and addressed as part of the deprescribing process (Scott et al., 2015). Importantly, within one patient, concerns and perceived necessity can differ between medications. A negative opinion on medication in general and willingness to reduce the number of medications does not necessarily imply that this patient is willing to stop the medication the HCP thinks is most appropriate to deprescribe. Aligning the assessment of the HCP with the beliefs of the patient is therefore essential. Identifying and addressing beliefs about medication of patients with limited medical knowledge can be a difficult task. To address these incomplete or incorrect beliefs about stopping medication, the use of benefit-risk communication tools similar to those that have been developed for prescribing decisions (Way et al., 2017) may be helpful. Beliefs about risks can be based on incorrect interpretation of information and are prone to confirmation bias. For the development of a deprescribing benefit-risk communication tool, making use of the mental model approach, which has been successfully applied in several nonpharmaceutical settings, could be helpful (Morgan et al., 2002). The mental model states that individuals construct a script about risks they perceive (Breakwell, 2014). This script is then used to estimate personal risk and adept behavior accordantly. In the mental model approach both correct and incorrect notions and beliefs about a certain risk in the target population are identified. Providing information that takes these initial beliefs and fears into account can help individuals to make better informed decisions.

When deprescribing cardiometabolic medication, it is important to acknowledge the role that treatment targets play in the process of initiating, continuing and/or intensifying medication treatment. Reducing HbA1c, blood pressure and cholesterol have been key treatment goals for patients with T2D and cardiovascular disease and may often be related to the need of being adherent to medication (Stratton et al., 2000; Selvin et al., 2004; Kirkman et al., 2018). When strict control is no longer expected to be beneficial or when it can even be considered harmful, new goals need to be set that are more fitting for the current situation. Involving patients in this process can support medication changes and reduce drug-related problems, as was shown in a study using goal attainment scaling during community pharmacist-led medication reviews (Verdoorn et al., 2019). In the future, HCPs might address this better also when initiating treatment, making it more clear that treatment targets should be personalized and adapted to the benefits and risks for the individual patient. Furthermore, monitoring the outcomes of deprescribing and explaining that slight increases in risk factor levels may be wanted can help the acceptance of deprescribing cardiometabolic medication. Also, having the option to restart when the outcomes are not as expected or wanted can be important for patients. Finally, when deprescribing is proposed by another HCP than the prescribing physician, clearly communicating to the patient that the prescribing physician will be consulted before a final decision is made can be important to establish trust.

## Conclusion

Patients’ barriers to and enablers of deprescribing cardiometabolic medication are in part similar to deprescribing of other medication groups and of younger patients. Four general typologies were identified regarding the patients’ willingness to stop medication but within one patient the willingness or resistance to deprescribing can be linked to specific medication. It is thus important to explore the patients’ attitudes in general and in relation to specific medication before proposing to deprescribe medication. Regarding cardiometabolic medication, the concept of personalized treatment targets needs to be explained so that it is clear that benefits and risks of such medication change when people get older. To monitor and evaluate the effects of reducing the medication assessing clinical measurements is helpful when patients understand that higher treatment targets are wanted. Our findings illustrate the complexity of decision making and the need for involving patients early in the process of deprescribing.

## Data Availability Statement

The datasets presented in this article are not readily available because this would be in conflict with the informed consent signed by the participants. Requests to access the datasets should be directed to p.denig@umcg.nl.

## Ethics Statement

The studies involving human participants were reviewed and approved by Medical Ethic Commission of VU Medical Center. The patients/participants provided their written informed consent to participate in this study. All procedures performed in this study involving human participants were in accordance with the ethical standards of the Medical Ethic Commission of VU Medical Center (FWA00017598) and with the 1964 Helsinki declaration and its later amendments.

## Author Contributions

SC, GB, JA, TB-B, JH, MB, MH, KT, and PD: research idea and study design. SC, GB, JH, and MH: Data acquisition. SC, GB, TB-B, and PD: analysis and interpretation. KT, MH, MB, JH, and PD: supervision or mentorship. All authors contributed to the article and approved the submitted version.

## Funding

An unconditional grant has been provided by The Royal Dutch Pharmacists Association (KNMP) in order to perform this research.

## Conflict of Interest

The authors declare that the research was conducted in the absence of any commercial or financial relationships that could be construed as a potential conflict of interest.
