# Supplementary material for: Barriers and Enablers of Older Patients to Deprescribing of Cardiometabolic Medication: A Focus Group Study
Source: Front Pharmacol. 2020 Aug 20;11:1268. doi: 10.3389/fphar.2020.01268 (PMC7468428; doi:10.3389/fphar.2020.01268)
Supplement: Supplementary file 1 [file DataSheet_1.pdf]

Barriers and enablers of older patients to deprescribing of cardiometabolic medication:
A focus group study. Crutzen S e.a. (2020)

Appendix I. Topic list

Introduction of the focus group

- Welcome to this group discussion
- Introduce the present researchers
- We will have a short break around 11 am. We will be finished at 12 am at the latest
- There are no wrong answer to the questions, all answers are good
- Discussion among each other is fine as long as you respect each other's opinions: Feel free to say you have a different opinion or that you agree with someone.
- Everything you hear here during the group discussion is confidential
- Because of the limited time someone might be cut off to ensure that all issues are discussed within the time limit
- A sound recording will be made so the researcher can listen to the discussion afterwards. Your name will not be mentioned anywhere

Introduction of deprescribing

- I shall introduce shortly the topic of this morning. This topic has been researched for some time now, however for you it might be a new topic. So feel free to ask clarification if I'm not fully clear about the topic.

- Today we will talk about lowering the medication for blood pressure, cholesterol and sugar in the blood. These medication are meant to reduce the long term risks of cardiovascular disease and the risk of getting diabetes related complications. This is very important and often more medication is prescribed over time.

- However medication also has disadvantages. They can cause side effects for instance. When someone is getting older the body changes. This can increase the risk of side effects. And for some people taking a lot of medication can be a burden.

- After a while the risk of medication outweigh the benefit for some older patients. In those cases it might be better to stop medication or to reduce the dosing. By doing this we want to prevent that you we get side effects in the future

- Doctors and pharmacists are doing this more often than they used to do. They feel the need to know how you feel about stopping or reducing medication so they can help you better with your medication.

- Did I explain well what we will be talking about?

Questions:

Question 1: Could you shortly tell something about the medication you or the person you care for uses?
(Every participants will be asked to tell something. The following questions will be discussed among the participants)

Question 2: Who among you is taking medication they would rather stop? Could you tell something about that?
- What are the reasons for wanting to stop?
- What are the reasons to keep using the medication?
- What do the others think about this?

Question 3: Has it ever happened that medication that you have been taking for a long time was stopped?
- How was that for you?
- Who proposed to stop the medication? (doctor, nurse practitioner, other health care professional)

Question 4: What would you think if a doctor would propose to reduce or stop your medication for blood pressure, your hart, cholesterol or diabetes?
- Which emotion would this evoke?
- What are your ideas or wishes about stopping medication?
- Does it matter which medication it concerns? (Diabetes/blood pressure)
-What if a different health care professional would propose to stop? (Nurse practitioner, community pharmacist, specialist)

Question 5: How would you like to be involved in the decision to stop medication?
- To what extend would you like to actively participate in the decision process?
- To what extend do you want to be involved in making the final decision?
- Would you want to have time to think about it?
- What would you like to know before stopping?
- With whom would you like to talk before making a decision?
- What do the care givers think about this?

Question 6: What could be reasons for you to stop or keep taking medication for blood pressure, your hart, cholesterol or diabetes?
(use a flip over to write down the pros and cons of stopping medication)
- Advantages of stopping medication
- Disadvantages of stopping medication
- Which consideration is the most important?
- What would be the conditions to stop?
- What kind of help would be necessary?

Question 7: Which role could your community pharmacist play in stopping or reducing medication for blood pressure, your hart, cholesterol or diabetes?
-Signaling problems, making a proposal for your doctor?
- Providing information on the advantages and disadvantages?
- Other support?
- Medication reviews?

Question 8: Which role could your other health care professionals like nurse practitioners or specialized diabetes nurses play in stopping or reducing medication for blood pressure, your hart, cholesterol or diabetes?
- Providing information on the advantages and disadvantages?
- Support?
- Check-ups?

Question 9: Are there any other factors that are important for you regarding stopping or reducing medication?
