# Supplementary material for: Barriers and Enablers of Older Patients to Deprescribing of Cardiometabolic Medication: A Focus Group Study
Source: Front Pharmacol. 2020 Aug 20;11:1268. doi: 10.3389/fphar.2020.01268 (PMC7468428; doi:10.3389/fphar.2020.01268)
Supplement: Supplementary file 2 [file DataSheet_2.pdf]

**Appendix II. Coding scheme**

| | |
|---|---|
| **Thematic coding:** | Barrier to deprescribing<br>Enablers to deprescribing<br>Role of the general practitioner<br>Role of the nurse practitioner<br>Role of the community pharmacist<br>Role of the medical specialist<br>Role of the patient |
| **Theoretical domain framework coding:** | Knowledge<br>Skills<br>Social/professional role/identity<br>Beliefs about capabilities<br>Beliefs about consequences<br>Motivation and goals<br>Memory, attention and decision processes<br>Environmental context and resources<br>Social influences<br>Emotion<br>Behavioural regulation<br>Nature of behaviour |
| **Attribute coding:** | Adverse events<br>Cardiovascular medication<br>Diabetes medication<br>Co-medication<br>Deprescribing<br>Medication review<br>General practitioner<br>Nurse practitioner<br>Community pharmacist<br>Medical specialist |
