# Supplementary material for: Barriers and Enablers of Older Patients to Deprescribing of Cardiometabolic Medication: A Focus Group Study
Source: Front Pharmacol. 2020 Aug 20;11:1268. doi: 10.3389/fphar.2020.01268 (PMC7468428; doi:10.3389/fphar.2020.01268)
Supplement: Supplementary file 3 [file DataSheet_3.pdf]

Barriers and enablers of older patients to deprescribing of cardiometabolic medication:
A focus group study. Crutzen S e.a. (2020)

Appendix III. Summary of barriers and enablers per patient

Focus group 1:
Patient 1:
Barriers: General practitioner does not initiate a conversation about stopping medication, you have to ask about it. The general practitioner is not always available.
Enablers: Would like to take less medication. Statins caused side effects in the past, which resulted in stopping the statins. Sees medication as "poison".

Patient 2:
Barriers: Does not possess the necessary knowledge to make decisions about her medication. Does not have enough information about treatment targets of clinical measurement.
Enablers: Medication is bad for you, would like to stop medication because of the negative consequences of medication.

Patient 3:
Barriers: Feels well on the medication he/she is taking. Has little interest in what happens to his/her medication.
Enablers: x

Patient 4:
Barriers: Feels well on the medication he/she is taking. Only trusts his/her cardiologist to chance medication.
Enablers: x

Patient 5:
Barriers: Does not trust the nurse practitioner to reduce medication. Is not invited by a community pharmacist for a conversation about medication. Considers stopping anticoagulants not possible because of a transient ischemic attack in the past.
Enablers: x

Patient 6:
Barriers: Because of the medication thing are going well health wise. It is only possible in hindsight to determine whether or not stopping medication caused problems. Does not have the knowledge to talk to the physician, the physician should make the decision about stopping medication.
Enablers: Would like to take less medication. Trusts physicians to reduce medication.

Patient 7:
Barriers: If you take a lot of medication then you do not know which one causes the side effect. Constant changes to medication are bothersome.
Enablers: Has successfully stopped with a ACE-inhibitor in the past. Would like to take less medication

Patient 8:
Barriers: Patient does not know enough about medication in order to make a decision about stopping medication.
Enablers: Does not want to take NSAIDs anymore because of a gastric bleed in the past. Does not see the need for statins.

Focus group 2:
Patient 1:
Barriers: x
Enablers: Does not see the need for statins. Would want to stop medication if clinical measurements are monitored and stopping is evaluated.

Patient 2:
Barriers: Currently satisfied with his/her health status.
Enablers: Would like to stop medication if clinical measurements are monitored and stopping is considered a test.

Patient 3:
Barriers: Changes can be difficult to handle and things are going well health wise. It is necessary to take medication.
Enablers: Does not like it when medication is added. Would be willing to stop medication in consultation with a physician.

Patient 4:
Barriers: Medication prescribed by the cardiologist is important. Does not trust the general practitioner or nurse practitioner to change medication.
Enablers: Does trust the cardiologist to stop medication.

Patient 5:
Barriers: Some medication is absolutely necessary.
Enablers: Willing to stop medication that is not absolutely necessary, especially medication with bothersome side effect such as oral corticosteroid and high ceiling diuretics.

Patients 6:
Barriers: Things are going well health wise, why change anything. Bad experiences with stopping medication in the past. Which causes the patient to be anxious about what would happen if medication is stopped.
Enablers: x

Patient 7:
Barriers: x
Enablers: x

Patient 8:
Barriers: The cardiologist does not listen. Had bad experiences with stopping medication, namely return of cardiac complains.
Enablers: x

Patient 9:
Barriers: Experienced return of complains after stopping medication in the past. Not actively looking to change medication when thing are going well health wise. It is hard to pin point the medication that is responsible for a certain side effect.
Enablers: Would like to take less medication. Had severe nausea in the past because of medication. Is willing to stop medication, especially in the presence of side effect. If thinks are not going well after stopping you can always restart.

Patient 10:
Barriers: Stopping medication is dangerous. You do not know which medication helps with what.
Enablers: Suffers from side effects. Would trust the physician if he/she would like to stop medication. you can always restart. Taking medication for long periods is bad for you, therefore he/she would like to stop medication to combat this. Monitoring is important after stopping.

Table III.1. Categorisation of the patients in three groups divided based on a positive, negative or indifferent attitude towards medication

Positive attitude towards medication	Negative attitude towards medication	Indifferent attitude towards medication
FC1, P4	FC1, P1	FC1, P3
FC1, P5	FC1, P2	FC2, P7
FC1, P6	FC1, P7	
FC2, P2	FC1, P8	
FC2, P3	FC2, P1	
FC2, P4	FC2, P5 Ambivalent	
FC2, P6	FC2, P9 Ambivalent	
FC2, P8	FC2, P10	

Table III.2. Categorisation of the patients in three groups divided based on being resistant to stop, willing to stop or being indifferent towards stopping

Resistant to stop	Willing to stop	Indifferent to stopping
FC1, P4	FC1, P1	FC1, P3
FC1, P5	FC1, P2	FC2, P7
FC2, P4	FC1, P6	
FC2, P6	FC1, P7	
FC2, P8	FC1, P8	
	FC2, P1	
	FC2, P2	
	FC2, P3	
	FC2, P5	
	FC2, P9	
	FC2, P10	
